# Treatment of Leptomeningeal Disease with Tepotinib in a Patient with Lung Adenocarcinoma Harboring MET Exon 14 Skipping Mutation Presenting with Extensive Metastasis Involving Duodenum

**DOI:** 10.3390/reports8020096

**Published:** 2025-06-18

**Authors:** Jacquelyn Shugarts, Aida Amado, Taylor Praska, Monica Camou, Jiaxin Niu

**Affiliations:** Department of Medical Oncology, Banner MD Anderson Cancer Center, Gilbert, AZ 85234, USAmonica.camou3@bannerhealth.com (M.C.)

**Keywords:** METex14 subtype, leptomeningeal, duodenal metastases

## Abstract

**Background and Clinical Significance:** The mesenchymal–epithelial transition (MET) exon 14 skipping mutation (METex14) is a rare genetic alteration occurring in non-small cell lung cancer (NSCLC). Tyrosine kinase inhibitors (TKIs) are the approved treatment for first-line therapy in a metastatic setting. However, the unusual presentation of gastrointestinal metastasis and leptomeningeal carcinomatosis (LMD) poses significant treatment challenges. **Case Presentation**: Here we report a case of a 72-year-old male with metastatic METex14-positive NSCLC, presenting with brain and duodenal metastases. **Conclusions**: The patient responded exceptionally well to first-line chemoimmunotherapy, achieving clinically complete remission for 2 years. He subsequently developed cerebellar metastasis and leptomeningeal disease (LMD) but demonstrated a remarkable response to tepotinib and continued to enjoy radiographic complete remission over 2.5 years at the time of this report.

## 1. Introduction and Clinical Significance

The mesenchymal–epithelial transition exon 14 (METex14) skipping mutation is an uncommon genetic alteration found in 3–4% of NSCLC [1]. Other than traditional chemotherapy, immunotherapy or chemoimmunotherapy combination has been widely used to treat advanced METex14-positive NSCLC [2,3]. Recently, the MET TKIs, capmatinib and tepotinib, both of which bind to the ATP-binding site of the MET kinase domain to inhibit downstream signaling in METex14-altered tumors, have been approved by the FDA in the US and quickly adopted as the first-line therapy in the metastatic setting [4]. Both gastrointestinal metastasis and leptomeningeal carcinomatosis are rare presentations of the advanced NSCLC including METex14 subtype [5,6,7]. Treatment options in this scenario remain limited.

Here we report a case of a 72-year-old male with metastatic METex14-positive left upper lobe adenocarcinoma, who presented with brain and duodenal metastases, achieved a great response to first-line chemoimmunotherapy with clinically complete remission for 2 years. The patient subsequently developed cerebellar metastasis and LMD, responded remarkably well to tepotinib and continued to enjoy the radiographic complete remission in the past 2 and half years at the time of this report.

## 2. Case Presentation

**Figure 1:**
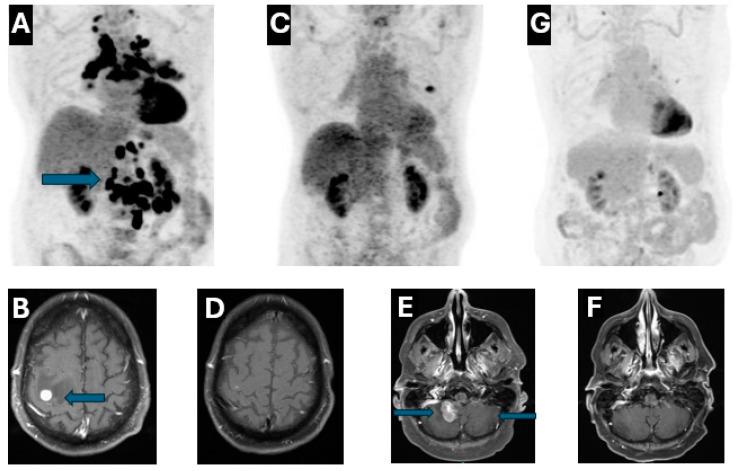
(**A**), *PET-CT*: Baseline tumor burden including duodenal metastasis (12/2019); (**B**), *MRI brain:* Brain metastasis at the diagnosis (12/2019); (**C**), *PET-CT*: 3 months after chemoimmunotherapy (4/2020); (**D**), *MRI brain*: 3 months after chemoimmunotherapy (4/2020); (**E**), *MRI brain*: Baseline with cerebellar metastasis and LMD (6/2022); (**F**), *MRI brain*: 6 weeks after chemotherapy and tepotinib (7/2022); (**G**), *PET-CT*: 15 months after re-starting tepotinib (6/2024).

A 72-year-old male, a former smoker with a five-year smoking history, originally presented to the emergency department (ED) with progressively worsening nausea, vomiting and cough with dyspnea in November 2019, computed tomography (CT) chest angiography showed a 1.7 cm spiculated nodule in the left upper lobe. CT abdomen and pelvis further demonstrated extensive metastasis to the mediastinal, cervical, retroperitoneal, and mesenteric lymph nodes. The patient reported melena and was noted to have severe anemia with hemoglobin to 6.7 g/deciliter which prompted an esophagogastroduodenoscopy (EGD) with biopsy of the third portion of the duodenum. Biopsy revealed poorly differentiated adenocarcinoma, positive for TTF-1 and Napsin A, highly consistent with lung origin, and ruling out other potential sites. Outpatient Positron Emission Tomography (PET)-CT confirmed the CT findings and demonstrated FDG-avidity in the duodenum, supporting the diagnosis of lung cancer with duodenal metastasis (Figure 1A). Subsequent Magnetic resonance imaging (MRI) Brain was ordered due to progressive left arm weakness, which confirmed the correlation of symptomatic brain metastases with four enhancing lesions measuring up to 1 cm (Figure 1B). He completed stereotactic radiosurgery (SRS) to brain metastases and regained full motor and sensory function in the left arm. Next-generation sequencing (NGS) revealed METex14 skipping deletion with a programmed death-ligand 1 (PD-L1) > 80%. Given the great tumor burden and lack of effective targeted options at that time, the patient received chemoimmunotherapy combination with carboplatin/pemetrexed/pembrolizumab for four cycles. Repeat imaging studies in April 2020 exhibited a remarkable response both intracranially and extracranially (Figure 1C,D).

The patient continued immunotherapy maintenance with pembrolizumab, with sustained response for almost two years until October 2020. The patient presented to the ED with left hand weakness and was diagnosed with a new left frontal solitary brain metastasis with significant vasogenic edema. The patient underwent craniotomy for tumor resection, confirming metastatic poorly differentiated adenocarcinoma of lung origin followed by SRS to the surgical bed in December 2020. In view of the isolated development of oligometastasis without extracranial progression, he continued on maintenance pembrolizumab until February 2022.

**Figure 2:**
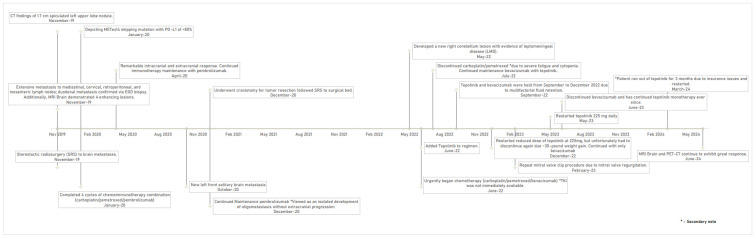
Timeline of clinical milestones over the 5-year treatment course.

The patient developed ataxia in May 2022, MRI Brain demonstrated a new lesion at the right cerebellum with evidence of leptomeningeal disease (LMD) (Figure 1E). He immediately began chemotherapy with carboplatin/pemetrexed/bevacizumab in early June 2022, as TKI was not immediately available. Tepotinib was added to the regimen approximately two weeks later. An MRI brain scan six weeks later showed an excellent therapeutic response with significant size reduction in the right cerebellar lesion and resolution of leptomeningeal enhancement (Figure 1F). At that time, carboplatin/pemetrexed was discontinued due to severe fatigue and cytopenia, while bevacizumab maintenance in combination with tepotinib was continued. The patient gained approximately 20 pounds despite the use of aggressive diuretics. CT chest/abdomen/pelvis in September 2022 revealed no evidence of disease, but development of mild pericardial effusion and bilateral pleural effusion. The fluid retention could be attributed to multiple factors including side effects of tepotinib, left mitral valve regurgitation due to chordal rupture and renal insufficiency. Thus, both tepotinib and bevacizumab were held from September to December 2022. The patient underwent two ultrasound guided left thoracenteses between November and December 2022 with pleural fluid cytology negative for malignancy. Bevacizumab/tepotinib was resumed in December 2022, but tepotinib was held again due to ~30-pound weight gain despite re-starting with a reduced dose at 225 mg daily. The patient underwent a repeat mitral valve clip in February 2023. With clinical improvement, tepotinib 225 mg daily was restarted in May 2023; both MRI brain and PET-CT in June 2023 demonstrated no evidence of disease. Bevacizumab was discontinued in June 2023, and the patient has remained on tepotinib monotherapy ever since. Of note, the patient ran out of tepotinib for three months due to insurance issues and was restarted in March 2024. The patient continues to have stable moderate fluid retention requiring compression stockings. Both MRI Brain and PET-CT continue to exhibit a great response without convincing evidence of progression of disease (Figure 1F,G). See Figure 2 for a timeline of clinical milestones over the 5-year course of treatment.

## 3. Discussion

Adenocarcinoma is the most common subtype of NSCLC, with approximately 60% of patients presenting with metastatic disease upon diagnosis [6]. The most common sites of metastasis include the liver, bones, adrenal glands, and the central nervous system (CNS). Up to 20% of patients manifest with brain metastases (BM) at the time of diagnosis, while nearly 10% of patients go on to develop LMD [8,9,10]. Metastasis to the GI tract is an uncommon presentation for a solid tumor. However, metastatic melanoma or lobular breast cancer are known to metastasize to the GI tract [7,11,12]. It is rare for primary lung adenocarcinoma to present with GI bleed due to metastasis, as seen in our case. METex14-positive lung adenocarcinoma was a recently recognized subtype of NSCLC, characterized by aggressive biological and clinical behavior. CNS metastases occur in approximately one-third of patients with METex14-positive lung cancer [6]. To our knowledge, GI metastasis has not been previously reported.

Before the development of effective TKIs against METex14, chemotherapy with or without immunotherapy was the mainstay treatment in advanced disease. As demonstrated in our case, the patient responded well to chemoimmunotherapy, achieving excellent disease control for nearly 2 years. The use of tepotinib was discussed with the patient as a future option, as it had not yet received FDA approval for METex14-positive NSCLC treatment at the time of the diagnosis. Additionally, due to the patient’s need for rapid symptom control, chemoimmunotherapy was selected as first-line therapy. Notably, his extracranial disease, including duodenal metastases, responded remarkably well without recurrence. However, he suffered from CNS recurrence, illustrating the ongoing challenge of long-term control of CNS metastasis in this patient population.

To date, two TKIs against METex14 mutation, capmatinib and tepotinib, have been approved in the US, with tepotinib demonstrating an overall response rate of 40–60% with a median duration of response of 11.1 months [13,14,15]. Although patients with treated brain metastases were included in clinical trials, those with symptomatic brain metastases were excluded. Therefore, there is no high-level evidence to support TKI use in this challenging setting. A single patient case study reported a great therapeutic response to tepotinib in a patient with symptomatic brain metastasis with LMD, suggesting a potential role for tepotinib in these scenarios [16,17]. Similarly, in epidermal growth factor receptor (EGFR) mutant lung adenocarcinoma, a TKI, osimertinib, demonstrated remarkable efficacy in patients with LMD, achieving a durable clinical response up to one year in the BLOOM study [18].

Our patient presented with symptomatic cerebellar metastasis and LMD. Given the urgent need for rapid disease control and symptom relief, we initiated combination therapy with chemotherapy (carboplatin/pemetrexed/bevacizumab) and tepotinib. The patient responded rapidly to the combination and continued to benefit from bevacizumab and tepotinib combination for almost a year. The addition of bevacizumab to his treatment plan was for its anti-VEGF (vascular endothelial growth factor) properties, aiming to reduce vascular permeability and mitigate peritumoral brain edema, which could have exacerbated his symptoms [19]. Although it is unlikely to tease out the exact contribution of tepotinib, the combination appeared to be safe and effective. Additionally, while there is scant evidence on the pharmacological rational for tepotinib’s CNS activity, in patient-derived xenograft models, after intravenous infusion the mean total tepotinib concentration was 2.87-fold higher in brain than plasma, suggestive of the excellent capability for tepotinib to cross the blood–brain barrier which could explain the given success in intracranial disease control [20]. Combining chemotherapy with TKIs has been extensively studied in EGFR mutant lung adenocarcinoma, with FLAURA 2 study demonstrating overall survival benefit when used in the frontline setting and MARIPOSA-2 study in the second line setting, respectively [21,22]. Prospective studies are needed to evaluate the combination of chemotherapy and tepotinib.

After discontinuation of bevacizumab, the patient remained on dose-reduced tepotinib monotherapy for 20 months despite a 3-month interruption, indicative of sustained CNS disease control. Nonetheless it is not without challenge to use either tepotinib alone or in combination with chemotherapy/bevacizumab. Fluid retention as an on-target adverse effect has been well documented previously [23]. In this unique case, it was confounded by cardiac dysfunction from mitral valve regurgitation and renal insufficiency. Increase in creatinine in patients on tepotinib may have resulted from creatinine retention, a unique side effect of tepotinib. His significantly reduced estimated GFR could be due to multiple factors such as heart failure, hypotension and baseline renal insufficiency as well as a direct effect of tepotinib [24]. The multidisciplinary team collaborated to manage tepotinib toxicity by having oncology adjust dosing for fluid retention, cardiology monitor and address mitral regurgitation via echocardiogram and valve clip procedure, and nephrology to optimize renal function amid cardiac and drug-related strain. Additionally, pharmacology to tailor dosing while minimizing drug interactions to minimize toxicity, especially considering the patient’s renal and cardiac complications. In turn, his cardiac function was restored and renal function gradually returned to baseline.

## 4. Conclusions

In conclusion, we report, to the best of our knowledge, the first case of duodenal metastasis with late onset LMD in a patient with NSCLC harboring METex14 mutation. The patient was successfully managed with systemic therapy, including chemoimmunotherapy, chemotherapy/tepotinib combination followed by tepotinib monotherapy and remains in radiographic remission 2 and half years after developing LMD. Further prospective studies are warranted in this population with LMD as there are limited ongoing or future clinical trials that specifically focus on METex14-positive NSCLC with CNS involvement, and many of these trials exclude patients with symptomatic CNS metastases. This exclusion is particularly concerning as it overlooks a significant subset of patients who may benefit from targeted therapies like tepotinib.

## Data Availability

The original contributions presented in this study are included in the article. Further inquiries can be directed to the corresponding author.

## Abbreviations

The following abbreviations are used in this manuscript:

MET	Mesenchymal–epithelial transition
METex14	Mesenchymal–epithelial transition exon 14 skipping mutation
NSCLC	Non-small cell lung cancer
TKIs	Tyrosine Kinase Inhibitors
LMD	Leptomeningeal Disease
ED	Emergency Department
CT	Computed Tomography
EGD	Esophagogastroduodenoscopy
PET	Positron Emission Tomography
MRI	Magnetic Resonance Imaging
NGS	Next-Generation Sequencing
PD-L1	Programmed Death-Ligand 1
BM	Brain Metastases
CNS	Central nervous system
EGFR	Epidermal Growth Factor Receptor
